# A new species of sack-bearer moth (Lepidoptera, Mimallonidae) from Florida

**DOI:** 10.3897/zookeys.1273.181781

**Published:** 2026-03-23

**Authors:** Ryan A. St Laurent, Scott Wehrly, Jeffrey Slotten

**Affiliations:** 1 Museum of Natural History, University of Colorado, Boulder, CO, USA & Department of Ecology and Evolutionary Biology, University of Colorado, Boulder, CO, USA Museum of Natural History, University of Colorado Boulder United States of America; 2 Tavares, FL, USA Unaffiliated Tavares United States of America; 3 McGuire Center for Lepidoptera & Biodiversity, Gainesville, Florida 32611, USA McGuire Center for Lepidoptera & Biodiversity Gainesville United States of America

**Keywords:** *
Cicinnus
*, conservation concern, endemic, Florida Scrub, Mimallonoidea, taxonomy

## Abstract

A rarely reported new species of Mimallonidae from Florida, USA, is described. Remarkably, the distinctive new species has not been seen across much of its historical range since the 1960s, and all records come from six somewhat isolated white sand Florida Scrub habitats, one of which is completely destroyed and most others clearly imperiled. DNA barcoding sequences from the disparate populations suggest notable genetic diversity in the few known populations, meaning extirpation of any of them could signal loss of critical population structure of an already narrowly endemic species. *Cicinnus albarenicolus* St Laurent, **sp. nov**. is compared to the only other similar species in North America, *C. melsheimeri* (Harris in Doubleday), a common denizen of oak forests throughout the eastern United States, including Florida, southern Canada, and the Rocky Mountains. Until its distribution and life history are more well-understood, *C. albarenicolus* will remain an enigmatic taxon of major conservation concern.

## Introduction

Mimallonidae is a relatively species-poor family of moths endemic to the Americas. Recent years have seen an active period of the systematic revision of the previously poorly studied family, resulting in over 320 species recognized in 44 genera classified using phylogenomics (St Laurent and Kawahara 2019; St Laurent et al. 2020b). Most species are found in South America with decreasingly fewer species northward into Central America, Mexico, the United States, and southern Canada. The United States is home to just five species. *Cicinnus melsheimeri* (Harris in Doubleday, 1841) and *Lacosoma chiridota* Grote, 1864 occur in the eastern half of North America (both also occurring scarcely in Canada and *C. melsheimeri* sporadically in the Rocky Mountains), *Lacosoma elassa* (Franclemont, 1973) in southern Texas, and two primary species in Arizona: *Cicinnus chambersi* St Laurent, Reeves & Kawahara, 2020a and *Lacosoma arizonicum* Dyar, 1898. Two of these five species, *C. chambersi* and *L. elassa*, are known from fewer than a dozen specimens and each was described in the last half century. Unsurprisingly, given the poor species diversity and some species being rather uncommonly collected, very little is known about the life history of the species occurring in the United States. Those with known host plants are exclusively associated with various Oaks (*Quercus* sp.).

*Cicinnus* Blanchard, 1852 contains 14 described species across its range in the Americas, with species occurring from southern Canada to southern Brazil and Argentina. The most familiar North American species, *C. melsheimeri*, is sister to all other species in the genus according to phylogenomic data, and is morphologically the most distinctive, bearing a pair of vincular arms on the male genitalia, which are absent in the rest of the genus (St Laurent et al. 2020b). *Cicinnus melsheimeri* is the most widespread mimallonid in North America, occurring along the entire eastern seaboard from Florida to Massachusetts, west to the plains, with various pockets of occurrence in southern Ontario, west Texas, and the southern Rocky Mountains of New Mexico, Arizona, Colorado, and into Mexico. Based on available molecular data, this wide distribution is represented by a single species, though morphological variation is particularly high, with considerable genitalia variation within populations and further differences in wing patterning, size, and shape across the geographic range. *Cicinnus melsheimeri* is exclusively associated with oaks and is usually restricted to sandy oak-pine barrens in the northeastern parts of its range and various oak woodlands westwards, whereas it occurs in most habitats farther south (Franclemont 1973; Wagner 2005).

Since his revision of North American Mimallonidae, Franclemont (1973) remarked that some Florida populations were somewhat different morphologically, with the apex of the forewing less “produced.” However, due to variation in male genitalia across the wide range of *C. melsheimeri* and unrecognized genitalia differences between males of this Floridian morphotype and other *C. melsheimeri* populations, including in Florida, the specific identity of the unique Florida *Cicinnus* has not been questioned. In Heppner’s (2003) Checklist of Florida Lepidoptera, one of these Floridian *Cicinnus* was figured to represent *C. melsheimeri*. Apart from Franclemont’s mention of the distinctive Florida population and Heppner’s figure, no further work has been conducted to determine whether this population could represent a distinct taxon. After reviewing most major North American collections and examining and sequencing mitochondrial DNA of nearly all available exemplars of this morphotype, it is clear that this population is distinct from *C. melsheimeri* and warrants species description. This is particularly timely given the apparent rarity and likely imperiled status of this new species, which is highly restricted in range within Florida, occurring only in white sand scrub habitat. Furthermore, it has not been seen for over 60 years in five of the six localities where it is known to occur and may be extant at only a single site. This study aims to formally describe this new species, figure it, and compare it to typical *C. melsheimeri* populations with the goal to bring attention to a rare species likely in need of protection and further research.

## Materials and methods

### Morphological and specimen data

Genitalia examination included *Cicinnus* slides prepared by Franclemont in the Cornell University Insect Collection (CUIC) and dissections prepared for the current study, following Lafontaine (2004) in methodology and St Laurent and Kawahara (2019) for terminology. *Cicinnus* genitalia are complicated structures, and so all new preparations are stored in glycerol-filled microvials as opposed to slide mounting, which flattens the otherwise three-dimensional structures. Labels of the holotype of the new species are given verbatim (except coordinates, see below), and other examined material, including paratypes, are presented in order of locality from North to South; alphabetically by county for *C. melsheimeri*. All wing measurements were made with electronic calipers accurate to 0.01 mm.

For consistency, the only specimens figured are those photographed with the same photographic equipment: a Nikon D7500 equipped with a Laowa 60 mm f/2.8 2X Ultra-Macro lens (f = 2.8), and a 5600K LED Macro Ring Light attached to the lens. This is due to the pink hue of the new species being difficult to ascertain, depending on variations in lighting. Specimens of *C. melsheimeri* were photographed from localities where the new species was also collected since the two *Cicinnus* species are locally sympatric, and *C. melsheimeri* from extra-Floridian localities are included to illustrate variation. For all specimen photos, multiple photos were taken at different focal lengths and stacked in Photoshop 2026 as part of Creative Cloud. Specimens were photographed on a white background with a gray card standard, and adjustments in exposure made in Photoshop where necessary. Genitalia were photographed with a Canon EOS 5D Mark II with a Macro Photo MP-E 65 mm f/2.8 1–5× Manual Focus Lens for EOS at 4.5 or 5X. The genitalia images were brightened, dust and other contaminants removed, and set in plates using Photoshop.

The following collections were examined for relevant material of the new species and *C. melsheimeri* from Florida, but not all *C. melsheimeri* specimens seen by the authors from global collections are reported in the present study, particularly outside of Florida:

**AMNH** American Museum of Natural History, New York, New York, USA;

**CUIC** Cornell University Insect Collection, Ithaca, New York, USA;

**CUMNH** University of Colorado, Boulder, Museum of Natural History, Boulder, Colorado, USA;

**MCZ** Museum of Comparative Zoology, Harvard University, Cambridge, Massachusetts, USA;

**MGCL** McGuire Center for Lepidoptera & Biodiversity, including the Florida State Collection of Arthropods (FSCA), Gainesville, Florida, USA;

**SWC** Scott R. Wehrly, personal collection, Tavares, Florida, USA;

**USNM** National Museum of Natural History [formerly United States National Museum of Natural History], Washington DC, USA

### Distribution data

For simplicity, detailed locality data for *C. melsheimeri* is only provided for Florida populations, considering that it can be found in much of the eastern United States and is quite common in institutional collections and on online community science platforms like iNaturalist (iNaturalist 2025) which, together, were used to build the distribution map within Florida. Some exemplars of *C. melsheimeri* from extra-Floridian localities are figured to show the range of variation in this species. Coordinates for data points and their respective sources are provided in Suppl. material 1. The map was constructed with SimpleMappr (Shorthouse 2010). We omit coordinates for the sole contemporary population given the potential sensitivity of the species, but this population is represented on the map. Implicit or non-written information gleaned from labels is presented in square brackets.

### Molecular data

For sequenced specimens (most specimens of new species, and many *C. melsheimeri*) the end of a given specimen’s data in the Material examined section includes the Barcode of Life Datasystems process ID (starting with “NOMIM” or “UCMCL”) followed by the source collection’s unique identifier in parentheses (the numbers in parentheses equate to the numerical values of the “sample ID” in BOLD), as shown in Suppl. material 2. Although genitalia, external morphological characters, and maculation are sufficient to recognize the newly described species and differentiate it from *C. melsheimeri*, additional confirmation of the specific identity of the new species was provided by phylogenetic analysis of available mitochondrial cytochrome *c* oxidase subunit I (COI), a commonly used biological “barcode” marker (Hebert et al. 2003). Publicly available COI barcodes of *C. melsheimeri* and as many other *Cicinnus* species as available were downloaded directly from the BOLD (Ratnasingham and Hebert 2007) and combined with new sequences of most known historical specimens of the new species (the specimens from MCZ, MGCL, and SWC were not sequenced). In total, 38 *Cicinnus* barcodes were newly sequenced for this work, 25 of *C. melsheimeri* and 13 of the new species. These records have also been submitted to GenBank with their accession numbers included in Suppl. material 2. Additionally, 27 *Cicinnus* barcodes were downloaded from BOLD. A single species of the related genus *Gonogramma* Boisduval, 1872, which belongs to the same tribe as *Cicinnus*, is used as an outgroup to root the tree (St Laurent et al. 2020b). Of the 66 total barcodes used in this study, all but seven samples had less than 3% missing data.

The molecular data were aligned with MUSCLE in AliView (Larsson 2014) with a final alignment length of 658 base pairs, and phylogenetic tree inference was carried out with IQ-TREE 3.0.1 (Minh et al. 2020; Wong et al. 2025). ModelFinder in IQ-TREE was used to identify the nucleotide substitution model (TIM2+F+G4) for the unpartitioned dataset during a single preliminary run. Using that model, one hundred independent IQ-TREE runs were performed (Kalyaanamoorthy et al. 2017), using 1000 UltrafastBootstraps and 1000 Shimodaira Hasegawa approximate Likelihood Ratio Test (SH-aLRT) as measures of support, with the -bnni command used to reduce inherent biases of UFBoot (Minh et al. 2013; Hoang et al. 2018). Within-group and between-group pairwise distances of COI sequences were calculated with Kimura 2-parameter model (Kimura 1980) in MEGA12 (Kumar et al. 2024). The fasta file alignment and the unrooted .tre file for the final tree figure are included as Suppl. materials 3, 4, respectively.

## Results

### Cicinnus albarenicolus

Taxon classificationAnimaliaLepidopteraMimallonidae

St Laurent sp. nov.

40F624CE-4970-5FC7-A957-2E901ECDA2C0

https://zoobank.org/4B4E75BD-14B0-4F2A-A4F7-20B856266E8F

Figs 1–4, 9, 10, 12, 13, 15

#### Type material.

***Holotype*. United States of America – Florida: Lake Co**.: ♂, “Cicinnus melsheimeri [sic] FL, Lake Co., Rt 455, nr Alexander Springs, Ocala National Forest, 64F, 25w black light bucket trap. 6-IV-2023. Scott R. Wehrly. MD / Barcode of Life DNA voucher specimen Sample Locator: CCDB-51558_A01 [blue label]/ St Laurent diss.: 2-13-26:1/ UCMC 0277792 [scannable barcode]/ Holotype ♂ *Cicinnus albarenicolus* St Laurent, 2026 [red label]/” (CUMNH). ***Paratypes***. (11 ♂, 7 ♀ total) all from **United States of America – Florida: Volusia Co**.: 1 ♀, Cassadaga, 1.V.1962, S. V. Fuller [collector] (MGCL 1031631); 1 ♀, as for previous but 12.V.1963, (MGCL 1031691); 1 ♀, Cass[adaga], Jn[?] 5.52 [presumably 1952, but month illegible], [separate labels reading “BB 145”, “5229”, and “SVF” the latter likely referring to S.V. Fuller, collector] (MGCL 1031695). **Lake Co**.: 1 ♂, Cassia, 11.V.1964, Taylor [collector], Collection of Bryant Mather, St Laurent diss.: 5-7-25:3, NOMIM011-25 (AMNH 00171344). 1 ♂, Rt 455, Alexander Springs, Ocala National Forest, 25w black light bucket trap, 15.IV.2020, Scott R. Wehrly, MD [leg.] (SCW). 1 ♂, as for previous but mercury vapor light, 13.IV.2019, Scott R. Wehrly, MD [leg.] (SWC). **Hernando Co**.: 1 ♂, Weeki Wachee Springs, 19.V.1955, J.F. May [collector], NOMIM001-25, Barcode of Life DNA voucher specimen Sample Locator: CCDB-51403_A01 (AMNH 00171338); 1 ♂, as for previous but 28.V.1960, St Laurent diss.: 5-7-25:1, Barcode of life DNA voucher specimen Sample ID: 47560-F01 (USNMENT 02001374). **Highlands Co**.: 1 ♂, Archbold Biol[ogical] Sta[tion], 5.IV.1959, J.G. Franclemont [collector], Genitalia Slide 5674 J.G. Franclemont, NOMIM007-25, Barcode of Life DNA voucher specimen Sample Locator: CCDB-51403_A07 (CUIC 00013048); 1 ♀, as for previous but 30.III.1959, no dissection, NOMIM009-25, Barcode of Life DNA voucher specimen Sample Locator: CCDB-51403_A09 (CUIC 000131050); 1 ♀, as for previous but 1.IV.1959, no dissection, NOMIM010-25, Barcode of Life DNA voucher specimen Sample Locator: CCDB-51403_A10 (CUIC 000131049); 1 ♂, as for previous but 5.IV.1959, no dissection, NOMIM008-25, Barcode of Life DNA voucher specimen Sample Locator: CCDB-51403_A08 (CUIC 000131051); 1 ♀, as for previous but V.1961, HE & MA Evans [collectors], no dissection, partly illegible label reading “*C. egenaria*?! Walker” (MCZ 00239807). **Martin Co**.: 1 ♂, Port Sewall, 28–31.III.1949, NOMIM003-25, Barcode of Life DNA voucher specimen Sample Locator: CCDB-51403_A03 (AMNH 00171340); 1 ♀, as for previous but 11–15.III.1950, L. J. Sanford [collector], St Laurent diss.: 5-9-25:1, NOMIM005-25 Barcode of Life DNA voucher specimen Sample Locator: CCDB-51403_A05 (AMNH 00171342); 1 ♂, as for previous but 16–20.III.1950, L. J. Sanford [collector], NOMIM004-25, Barcode of Life DNA voucher specimen Sample Locator: CCDB-51403_A04 (AMNH 00171341); 1 ♂, as for previous but 19–23.III.1950, L.J. Sanford [collector], NOMIM002-25, Barcode of Life DNA voucher specimen Sample Locator: CCDB-51403_A02 (AMNH 00171339); 1 ♂, as for previous but 23–27.III.1950, L. J. Sanford [collector], NOMIM006-25, Barcode of Life DNA voucher specimen Sample Locator: CCDB-51403_A06 (AMNH 00171343). Paratypes with the following yellow label: Paratype ♂/♀ *Cicinnus albarenicolus* St Laurent, 2026.

**Figures 1–8.**
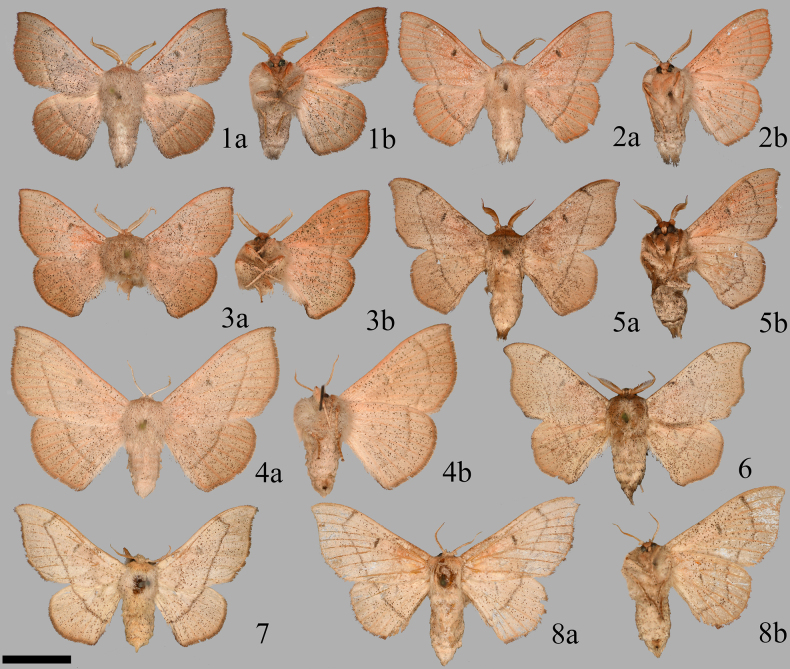
Florida *Cicinnus*, (**a**) or no letter denotes dorsum, (**b**) ventrum. **1**. *C. albarenicolus* male holotype, Ocala National Forest (UCMC 0277792; CUMNH); **2**. *C. albarenicolus* male paratype, Cassia, Florida (AMNH 00171344); **3**. *C. albarenicolus* male paratype, Archbold Biological Station (CUIC 00013048); **4**. *C. albarenicolus* female paratype, Archbold Biological Station (CUIC 000131049); **5**. *C. melsheimeri* male, Cassia (AMNH 00171346); **6**. *C. melsheimeri* male, Archbold Biological Station (USNMENT 02001410); **7**. *C. melsheimeri* female, Florida (AMNH); **8**. *C. melsheimeri* female, Weeki Wachee Springs (AMNH 00171345). Scale bar: 1 cm.

#### Diagnosis.

The only other species resembling *C. albarenicolus* anywhere in North America is its congener *C. melsheimeri*, which is more variable (over its entire geographic distribution) than *C. albarenicolus* and is a slightly larger species. Male mean forewing length differs significantly between the two species (*C. melsheimeri*: 17.73 mm, n = 31; *C. albarenicolus*: 16.06 mm, *N* = 10; Welch’s t-test: df = 22.93, p = 8.55 × 10^-5^). *Cicinnus albarenicolus* is decidedly pinker in hue, with shorter, broader wings and less deeply acute forewing apices than any population of *C. melsheimeri*. *Cicinnus melsheimeri* may also be more densely speckled and with more well-defined wing maculation, but this is primarily in extra-Floridian populations. Antemedial lines are absent in both *Cicinnus* species, but usually *C. melsheimeri* has a faint antemedial splotch on the costa, which is absent in *C. albarenicolus*. In general, *C. melsheimeri* from Florida are a bit smaller overall than those from the rest of North America (compare Floridian *C. melsheimeri* in Figs 5–8 to *C. melsheimeri* from elsewhere in Figs 17–22), paler in coloration and with less sharply acute forewing apices, but no Florida *C. melsheimeri* populations correspond to the diagnosis of *C. albarenicolus*. An important and highly consistent trait is the male antennae, which have a gradually shortened pectination length toward the apex in *C. albarenicolus*, whereas in *C. melsheimeri*, the distal quarter of the antennae has abruptly shorter pectinations. In both species, the pectinations gradually increase in length from the head to about half the antennal length, after which the pectinations gradually shorten distally toward the apex; the distinction between the two species is therefore most obvious in the terminal quarter of the overall antennal length.

**Figures 9–14.**
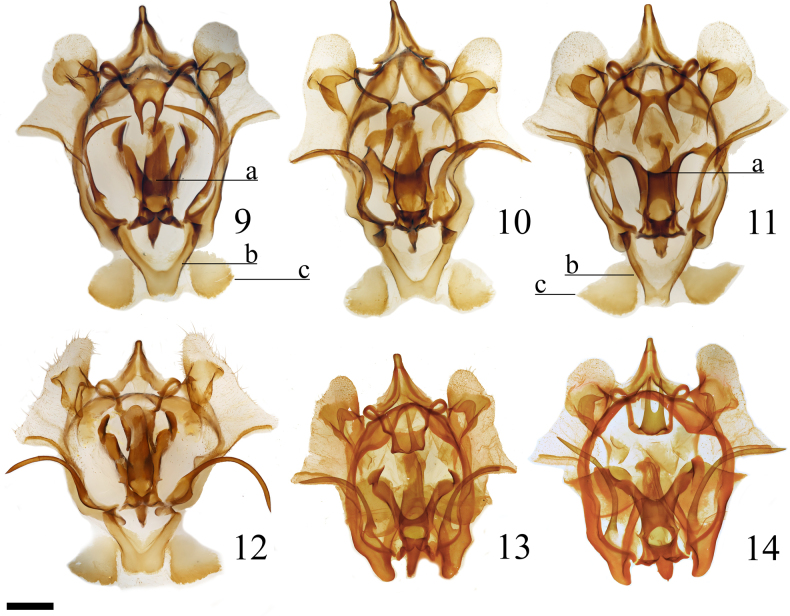
*Cicinnus* male genitalia, (**a**) denotes juxta center, (**b**) vinculum basal portion, (**c**) vinculum/VIII membranous portion. **9**. *C. albarenicolus* paratype, Cassia, vinculum unfolded, St Laurent diss.: 5-7-25:3 (AMNH); **10**. *C. albarenicolus* paratype, Weeki Wachee Springs, vinculum unfolded, St Laurent diss.: 5-7-25:1 (USNM); **11**. *C. melsheimeri* Cassia, vinculum unfolded, St Laurent diss.: 5-7-25:4 (AMNH); **12**. *C. albarenicolus* holotype, Ocala National Forest, vinculum unfolded, St Laurent diss.: 2-13-26:1 (CUMNH); **13**. *C. albarenicolus* paratype, Archbold Biological Station, vinculum folded, Genitalia Slide 5674 J.G. Franclemont (CUIC); **14**. *C. melsheimeri* New Jersey, vinculum folded, Genitalia Slide 5673 J.G. Franclemont (CUIC). Scale bar: 1 mm.

**Figures 15, 16.**
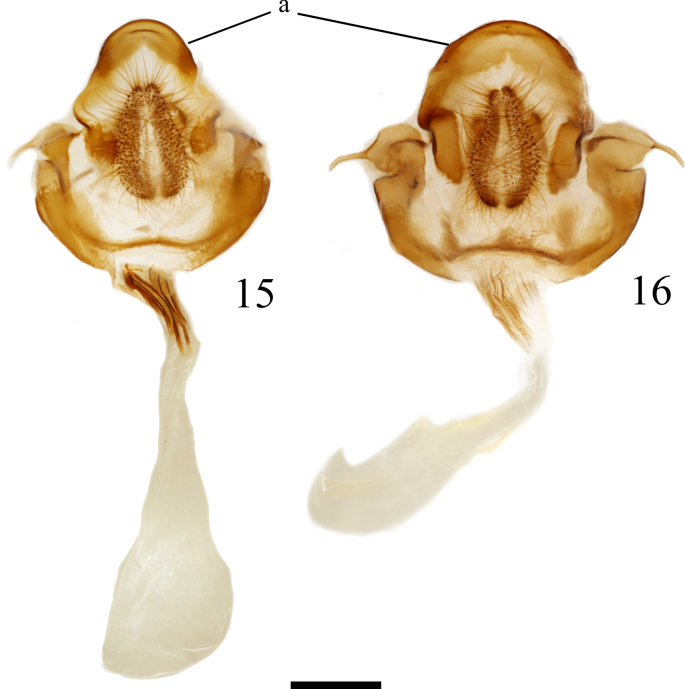
*Cicinnus* female genitalia, (**a**) denotes the VIII dorsal process. **15**. *C. albarenicolus* paratype, Port Sewall, St Laurent diss.: 5-9-25:1 (AMNH); **16**. *C. melsheimeri* Weeki Wachee Springs, St Laurent diss.: 5-9-25:2 (AMNH). Scale bar: 1 mm.

**Figures 17–22.**
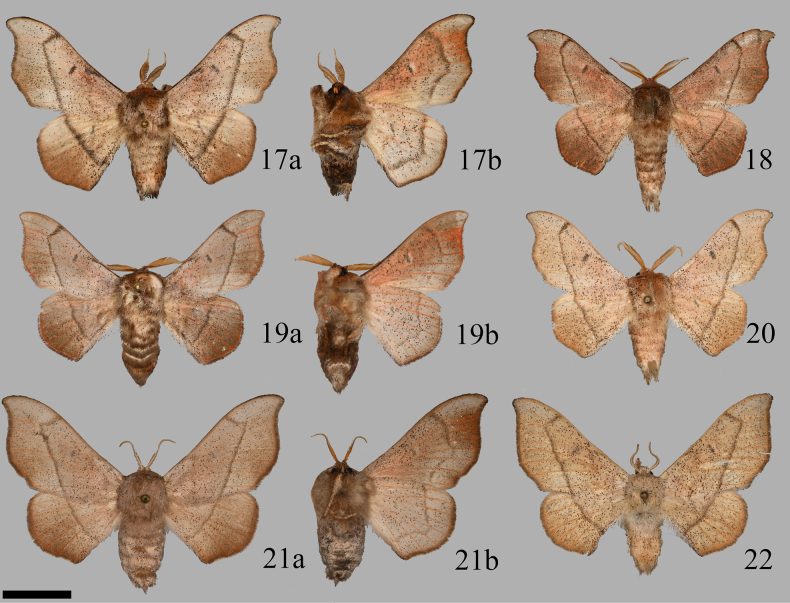
*Cicinnus melsheimeri* from extra-Floridian locales, (**a**) or no letter denotes dorsum, (**b**) ventrum. **17**. Male, USA, Tennessee (USNM); **18**. Male, USA, New York (CUIC); **19**. Male, USA, New Jersey (USNM); **20**. Male, USA, west Texas (USNM); **21**. Female, USA, New Jersey (CUIC); **22**. Female, USA, west Texas (CUIC). Scale bar: 1 cm.

Male genitalia are very similar in both species, as is typical within *Cicinnus* species-groups and more broadly in Cicinninae when comparing closely related species. However, the following differences are consistent between the two species, particularly when comparing sympatric populations: the curled tips of the juxta processes are wider, and the depth between them is deeper in *C. albarenicolus* (Fig. 9a) than in *C. melsheimeri* (Fig. 11a), and the claspers are slightly more projected in *C. albarenicolus* than in those of *C. melsheimeri* (this character is difficult to illustrate in two-dimensional images). The vinculum differs in the two species as well, with the bifurcated basal portion being thicker and more robust in *C. albarenicolus* (Fig. 9b) compared to the thinner and less stout appearance of the vinculum in *C. melsheimeri* (Fig. 11b), vincular thickness is especially evident in the holotype (Fig. 12). The weakly sclerotized distal component of this structure (which is of uncertain homology, it could be a modified part of the vinculum or part of VIII since it is embedded in intersegmental membrane) is rounded marginally in *C. albarenicolus* (Fig. 9c) and is more pointed in *C. melsheimeri* (Fig. 11c). In the holotype (Fig. 12) this character is less pronounced. Although difficult to verify from dissections, *C. albarenicolus* appears to have more pronounced curvature in the vincular arms than, at least, Floridian *C. melsheimeri*. Further minor differences are present in the uncus, gnathos, and tegumen, but these are less consistent and less discrete, and one should primarily rely on the aforementioned characteristics for discriminating between these two species based solely on male genitalia. For example, see gnathos variation in *C. melsheimeri* populations in Figs 11, 14. External appearance remains the simplest way to differentiate these two species.

Female genitalia of *C. albarenicolus* are recognizable by the dorsal projection of VIII, which is longer than wide (Fig. 15a), being somewhat tongue-like, and quite narrow compared to that of *C. melsheimeri*, which is wider than long and very broad in comparison (Fig. 16a). Apophyses anteriores are short in *Cicinnus*, but decidedly so in *C. albarenicolus* and appear to be shorter than in any observed *C. melsheimeri* dissections.

*Cicinnus melsheimeri* and *C. albarenicolus* are the only members of their respective clade, which is sister to the remainder of *Cicinnus*, a group of somewhat similar moths but which lack the vincular arms that are diagnostic of *C. albarenicolus* and *C. melsheimeri*. Populations of *C. melsheimeri* from Mexico may represent a third species of this clade, but these populations are overall quite similar to *C. melsheimeri* from the United States and Canada. *Cicinnus albarenicolus* is sister to Mexican *C. melsheimeri* + typical *C. melsheimeri*.

**Figure 23.**
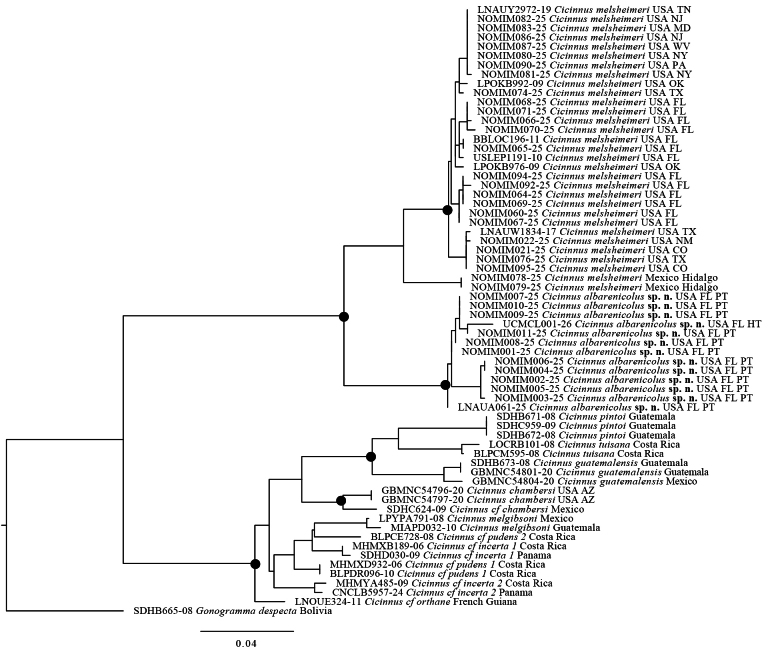
COI barcode gene tree inferred in IQ-TREE. Intraspecific support values not shown due to lack of space between all tips, see Suppl. material 4 for complete supports; other support values not shown if below 80 SH-aLRT and 95 UFBoot, black circles on nodes denote values higher than 80 SH-aLRT and 95 for UFBoot. The tree is rooted to *Gonogramma despecta*, remaining species are members of *Cicinnus*. Tips are labeled according to BOLD process ID followed by taxon name, country, type status (HT = holotype, PT = paratype), and if from USA, state (FL = Florida, CO = Colorado, TX = Texas, NM = New Mexico, OK = Oklahoma, NY = New York, PA = Pennsylvania, MD = Maryland, WV = West Virginia, NJ = New Jersey, TN = Tennessee). Percent genetic difference shown at bottom of figure.

Based on COI barcodes, *C. melsheimeri* and *C. albarenicolus* are clearly distinguished phylogenetically (Fig. 23) and by pairwise molecular distance. When comparing between-group mean distance of *C. melsheimeri* and *C. albarenicolus* the value is 6.68%. Within-group mean distance of all sampled *C. melsheimeri*, including Mexican samples, is 1.27% and 1.87% within *C. albarenicolus*.

#### Description.

**Male. *Head***. Coloration salmon pink. Antennae pale yellow, bipectinate to tip with apical pectinations notably shorter than remainder of antenna but gradually decreasing in length along distal quarter. Eyes large, covering more than two-thirds head area. Labial palpus very short, not extending beyond frons. ***Thorax***. Thickly scaled, coloration beige with dark brown petiolate scales irregularly covering area. Leg coloration mostly as for thorax basally but much pinker distally, tibial spurs very small, no longer than one-third length of first tarsomere. ***Forewing dorsum***. Forewing length 14.85–17.31 mm, avg. 16.06 mm, *N* = 10. Somewhat triangular, apex slightly falcate, but wing margin mostly convex, rounded. Ground color beige with perfuse salmon pink hue overall, especially antemedially, becoming more uniformly beige submarginally. Wing veins with pink scales. Overall wing covered in somewhat uniform speckling of dark brown petiolate scales. Discal cell marked distally by brown, narrow ovoid spot. Postmedial line faint, straight along entire length until perpendicularly angled or smoothly curving toward costa after passing Rs4. Antemedial line absent. ***Forewing ventrum***. Coloration and patterning similar to forewing dorsum, but more uniformly pink, markings less well-defined overall, antemedial line always absent, postmedial line convex, not angled toward costa. Retinaculum absent. ***Hindwing dorsum***. Rounded, coloration following similar pattern to dorsum but postmedial line very faint and convex, antemedial line absent, and discal mark faint. ***Hindwing ventrum***. Follows similar pattern as forewing ventrum, markings very faint, frenulum not visible but extremely reduced in *C. melsheimeri*, requiring wing removal to be observed (not performed here), and so may be present but vestigial. ***Abdomen***. Scale coloration as for thorax, stout. ***Genitalia***. (Figs 9, 10, 12, 13), *N* = 4. Complex, very similar to *C. melsheimeri*. Vinculum ovoid with inwardly directed, blunt ventral apodemes. Tegumen not well-defined as separate from vinculum. Uncus triangular with inwardly sloped lateral margins terminating in short blunt apex. Gnathos originating between base of uncus and anterior portion of tegumen/vinculum, looping upward initially and then swooping mesally with flat central portion terminating in pair of fingerlike projections that extend upward to just below uncus/vinculum junction. Valvae mostly membranous, most heavily sclerotized costally near junction with vinculum, sclerotization projected outward as triangular flap-like mesal extension, outer margin of valvae somewhat straight-margined but projected outward centrally where saccular margin curled inward forming a narrow channel housing curved vincular arms originating from vinculum on either side of phallus. Juxta fused to phallus with pair of sclerotized projections projected upward and away from phallus, tip of juxta projections distally twisted. Phallus cylindrical, distally membranous with narrow coecum phallus. Vesica bag-like. Base of vinculum (or as part of VIII tergite) extends outward as bilobed, sclerotized plate naturally held pressed upward covering genitalia. **Female. *Head***. As for male but with much shorter pectinations along length, pectinations gradually decrease in length from base to tip of antenna. ***Thorax***. As for male, but somewhat lighter. Legs as for male but tibial spines somewhat longer. ***Forewing dorsum***. Forewing length: 18.26–20.68 mm; *N* = 3. As for male, but wing shape slightly wider and paler pink overall, postmedial line may be more well-defined and slightly wavier, antemedial line absent. ***Forewing ventrum***. Similar to dorsum, markings very faint. ***Hindwing dorsum***. Following similar pattern to forewing dorsum, postmedial line somewhat curved, not angled toward costa, discal spot faint. ***Hindwing ventrum***. Follows similar pattern as forewing ventrum, postmedial line convex, discal spot nearly absent. ***Abdomen***. As for male, but somewhat lighter, broader, and longer, coloration as for thorax. ***Genitalia***. (Fig. 15), *N* = 1. Tergite VIII forms smooth, well-sclerotized, posteriorly projected tongue-like extension, length of extension greater than width. Apophyses anteriores roughly one-third length of apophyses posteriores, much thicker, stouter, more curved. Lamella antevaginalis membranous, lamella postvaginalis a narrow band of sclerotization. Ductus bursae with longitudinal sclerotized creases proximally, becoming membranous and not differentiated from bag-like corpus bursae. Papillae anales narrow, somewhat wider ventrally and mesally, apically forming blunt point, overall densely covered in elongate setae.

#### Etymology.

*Cicinnus albarenicolus* is named for the white sand scrub habitat of Florida, to which this species is endemic. The name is constructed from the combination of *albus* (white), *arena* (sand), and suffix -*icola/us* (dweller) from Latin. The name is a masculine noun.

#### Life history.

Nothing is definitively known about the life history of this new species except that it flies exclusively during the spring (March-May) and its inferred habitat is Florida Scrub (see Distribution below). Presumably, *C. albarenicolus* feeds on oaks like other North American Mimallonidae, but this must be confirmed. The range of this new species overlaps the ranges and habitats of characteristic oaks of scrub habitats: Myrtle Oak (*Quercus myrtifolia* Willd.) and Florida Scrub Oak (*Quercus inopina* Ashe); but *C. albarenicolus* ranges farther north than *Q. inopina* and is absent from the northerly part of the range of *Q. myrtifolia*. *Cicinnus melsheimeri*, on the other hand, is widespread in Florida (and beyond the state) and can be expected anywhere where oaks occur, and it conversely has a spring and summer brood. In 2020, the first author observed many unidentified *Cicinnus* larvae on *Q. myrtifolia* in an ideal scrub habitat in the Seminole State Forest, near Ocala and Cassia, where *C. albarenicolus* has been collected. However, *C. melsheimeri* occurs in the same habitats and has been collected at four of the six sites of *C. albarenicolus*, and so rearing to adulthood will be necessary to confirm identification until larval differences are identified.

#### Distribution.

**Figure 24.**
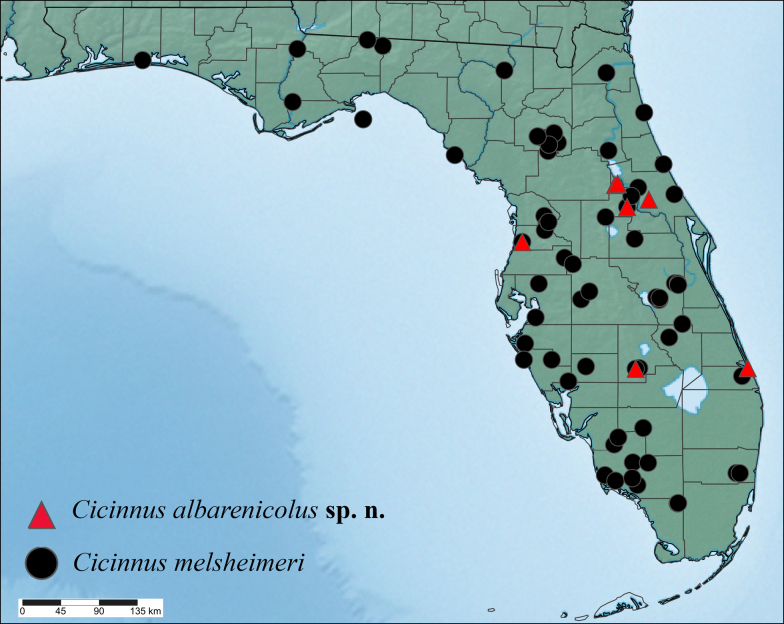
Distribution map showing both *Cicinnus* species in Florida. Localities include material examined by the authors and records of *C. melsheimeri* from iNaturalist.org. *Cicinnus melsheimeri* occurs widely outside of Florida, but those records are not shown. Both species occur where the red triangles and black circles overlap.

(Map in Fig. 24) *Cicinnus albarenicolus* is so far known only from six distinct sites in peninsular Florida: eastern Ocala National Forest, Weeki Wachee north of Tampa, Cassia and Cassadaga northeast of Orlando, the Archbold Biological Station on the Lake Wales Ridge in Central Florida, and coastal southeast Florida in Port Sewall. All of these localities have extant or historic rare Florida Scrub habitat, specifically white sand, open canopy scrub, which seems to be required by *C. albarenicolus*. This relict habitat formed on ancient sand dunes and is well-defined by unique flora and fauna, including other Lepidoptera (Hayden and Dickel 2015). This new species is not known from other, larger yellow sand scrub habitats like those of other regions of the Ocala National Forest (US Fish and Wildlife Service 1999), which can be inferred from the lack of any exemplars from that region after intensive insect collecting there for many decades.

#### Conservation.

Examination of satellite imagery of each of the confirmed localities that currently or historically supported *C. albarenicolus* shows small patches of white sand scrub habitats still exist in parts of Ocala National Forest, Weeki Wachee, southeast of Cassia in Seminole State Forest, and a very small example in Cassadaga; such habitat is still well-preserved in Archbold, though surrounding similar habitat is lost or degraded. The Port Sewall location has been completely developed and is unlikely to support this species contemporarily. With the exception of a trio of nearby sites in Ocala National Forest, the new species has not been seen from any of the other locations, including the intensively studied and preserved Archbold Biological Station, since the 1950s and 1960s. It is vital to conduct focused surveys during the known flight period in order to rediscover this exceedingly rarely seen moth.

Adding to the list of rarely seen North American Mimallonidae (*C. chambersi* is known from just 11 specimens and *Lacosoma elassa* from two), *C. albarenicolus* is likely of conservation concern. Unlike *C. chambersi* and *L. elassa*, which are considered Mexican taxa that barely extend to the United States and are presumably more widespread south of the US border, *C. albarenicolus* is endemic to peninsular Florida. It is restricted to imperiled habitats within this state.

### Cicinnus melsheimeri

Taxon classificationAnimaliaLepidopteraMimallonidae

(Harris in Doubleday, 1841)

8648D325-25DC-5ACF-8313-C74230711DE7

Figs 5–8, 11, 14, 16, 17–22

#### Florida material examined.

(77 ♂, 9 ♀ total) **USA: Florida: No County** • 3 ♀, St Laurent diss.: 5-9-25:3 (AMNH). **Alachua Co**. • 1 ♂, Gainesville, 12.IX.1961, H.A. Denmark (MGCL 1031744). • 2 ♂, 22.VIII.19.56, 13.V.195[illegible], H.A. Denmark, (MGCL 0131730, 1031741). • 1 ♀, 24.IV.1957, H.A. Denmark, (MGCL 1031710). **Citrus Co**. • 1 ♀, 8 mi. W. of Floral City, m-11, 17.V.1986, Dow (MGCL 1031689). **Collier Co**. • 1 ♂, Copeland, 20.VIII, H. Flaschka (MGCL 1031723). • 6 ♂, Collier Seminole State Park, 2.VIII.1986 (3 ♂), XI.1983 (1 ♂), 16.XI.1985 (2 ♂), Linwood C. Dow (MGCL 1031684, 1031687, 1031705, 1031709, 1031717, 1031719). • 1 ♂, Seminole State Park, 17–21.III.1990, H. Flaschka (MGCL 1031712). • 2 ♂, Collier Seminole State Park, 11.XI.1982, H.D. Baggett (MGCL 1031720, 1031722). • 2 ♂, Fakahatchee, 29.IV.1986, Dow (MGCL 1031707, 1031721). **Duval Co**. • 1 ♂, Jacksonville, Fla. Jr. Coll. S. Campus, X.VI.1980, Charles M. Stevens (MGCL 1031752). • 1 ♂, Jacksonville, Jax. Police Acad., 6.VI.1980 (MGCL1031728). **Hernando Co**. • 1 ♀, Weeki Wachee Springs, 5–30.V.1955, J.F. May, St Laurent diss.: 5-9-25:2, NOMIM066-25 (AMNH 00171345). • 1 ♂, McKethan Lake Rec. Area, Withlacoochee St. Forest, 23.IV.1983 (MGCL 00171345). • 2 ♂, McKethan Lake, 18.IX.1982, H.D. Baggett (MGCL 1031735, 1031750). • 2 ♂, Withlacoochee State Park, 17.VIII.1982, 26.IV.1991, W.L. Adair (MGCL 1031731, 1031733). **Highlands Co**. • 1 ♂, Parker Island, near Archbold Biological Sta., 31.III.1962, D.C. Ferguson, USNM-Mimal: 2779, NOMIM069-25 (USNM 02001412). • 1 ♂, Archbold Biological Sta., 2.IV.1962, D.C. Ferguson, USNM-Mimal: 2781, St Laurent diss.: 5-7-25:2, NOMIM067-25 (USNM 02001410). • 1 ♂, Archbold Biol. Sta., Lake Placid, 4.IV.1959, J.G. Franclemont, Genitalia Slide 5675 J.G. Franclemont, NOMIM065-25 (CUIC 00131052). **Hillsboro Co**. • 1 ♀, U. of So. Fl. Bait trail, 17.III.1982, W.L. Adair (MGCL 1031718). **Lake Co**. • 28 ♂, 1 ♀ Tavares, Acorn Circle, Squirrel Point, 22.9 m, 13.V.2013, 21.IX.2014, 24.IX.2014, 16.III.2015, 20.VIII.2015, 29.III.2016, 17.IV.2016, 23.IV.2017, 2.IV.2019, 2.IV.2020 (2♂), 10.IV.2021, 11.IV.2023, 12.IV.2023, 16.IV.2023 (2♂), 20.IV.2023, 21.IV.2023, 23.IV.2023, 25.IV.2023, 27.IV.2023 (2♂), 2.V.2023, 12.V.2023, 19.IV.2023, 22.IV.2023, 28.IV.2023 (2♂), 27.IX.2025, Scott R. Wehrly, MD [leg.], black/MV lights (SWC). • 1 ♂, Cassia, 1.V.1964, Taylor [collector], Collection of Bryant Mather, St Laurent diss.: 5-7-25:4, NOMIM071-25 (AMNH 00171346). • 1 ♂, 1.IV.1986 (MGCL 1031737). • 1 ♂, Forest Hills, 11.IV.1986 (MGCL 1031734). **Leon Co**. • 1 ♂, Miccosukee, 15.V.1982, W.L. Adair (MGCL 1031743). • 1 ♂, Tall Timbers Res. Sta., Lk. Iamonia, 19–21.V.1986, J.B. Heppner (MGCL 1031708). **Liberty Co**. • 2 ♂, Torreya State Park, 25.V.1980, 19.VIII.1982, H.D. Baggett (MGCL 1031711, 1031740). • 1 ♂, Torreya State Park, 16.VIII.1960, S.V. Fuller (MGCL 1031736). **Manatee Co**. • 2 ♂, Oneco, 27.III.1954, 31.III.1954, J.G. Franclemont, [process ID for CUIC specimen 000131054: NOMIM063-25; 000131053: NOMIM064-25] (CUIC 000131053, 000131054). **Marion Co**. • 1 ♂, 10-3-05, T. Miller (MGCL 1031747). **Miami-Dade Co**. • 1 ♂, Biscayne Bay (AMNH). • 1 ♂, Royal Palm Park, 4–10.XII.1938, NOMIM060-25 (AMNH 00171348). **Orange Co**. • 1 ♂, Orlando, 26.IV.1939, NOMIM092-25 (AMNH 00171347). **Okaloosa Co**. • 2 ♂, Shalimar, 18.IV.1964, 4.VI.1965 [H.O. Hilton coll.] (MGCL 1031748, 1031754). • 1 ♂, Ocean City, 17.VIII.1963, H.O. Hilton (MGCL 1031724). **Putnam Co**. • 1 ♂, University Reserve, Welaka, 10.IV.1962, D.C. Ferguson, USNM-Mimal: 2780, NOMIM068-25 (USNM 02001411). • 2 ♂, Weems Property, Red Water Lake, 29.VII.1967, 11.X.1967, H.V. Weems Jr (MGCL 1031747, 1031749). **Sarasota Co**. • 1 ♂, Siesta Key, 17.XI.1957, C.P. Kimball, NOMIM094-25 (AMNH 00171349). • 1 ♂, 21.V.1946, C.P. Kimball (MGCL 1031727). **St. Johns Co**. • 1 ♂, St. Aug[ustine] Lite[Lighthouse], XI.I.35, E.M. Davis (MGCL 1031753). **Volusia Co**. • 1 ♀, Tomoka State Park, 22–25.V.2000, J.B. Heppner (MGCL 1031751). • 1 ♂, New Smyrna, 16.X.1961, G.W. Rawson, USNM-Mimal: 2796, NOMIM070-25 (USNM 02001413).

#### Other dissections examined.

**USA: New Jersey: Ocean Co**. • 1 ♂, 1 ♀, Lakehurst, Franclemont diss.: 5673 (♂), 5708 (♀) (CUIC). **Indiana: Brown Co**. • 1 ♀, Brown County State Park, 12.VI.1979, D. L. Eiler, St Laurent diss.: 5-11-19:1 (MGCL). **Ohio: Geauga Co**. • 1 ♂, Thompson Township, ½ Mi. S. of jct. S.R. 528 & S.R. 66 U.S. Rt. 322, Warner Hollow Rd., 14.VI.1986, V.P. Lucas, St Laurent diss.: 8-10-18:1 (MGCL). **Texas** • 2 ♂, Big Bend, Franclemont diss.: 6405, 6408 (CUIC).

#### Remarks.

As stated earlier, *C. melsheimeri* is a common and widespread North American moth and having examined hundreds of specimens from across its range, only Floridian material is reported here, as it is the most relevant to the study, though we reference some extra-Floridian specimens for their available dissections. The present study is the first to look at COI barcode sequence diversity across the wide range of this species, and it is clear that some populations, particularly in Mexico and the western United States (west Texas, New Mexico, and Colorado) are well-differentiated and moderately differentiated phylogenetically, respectively, from the rest of the more eastern North American populations. Further work could explore the phylogeography of *C. melsheimeri* or cryptic diversity across the species. What is clear, however, is that *C. albarenicolus* is sister to all sampled populations of *C. melsheimeri* across North America (including Mexico).

## Discussion

From a phylogenetic context, it is clear that *C. albarenicolus* is divergent from all other *C. melsheimeri* populations, including the Mexican population, which itself is likely an undescribed species. Mimallonidae-wide phylogenomic studies and COI barcoding in the present study (Fig. 23) consistently recover *C. melsheimeri* as sister to all other *Cicinnus*, and so to have a deeply divergent lineage in Florida could impact biogeographic reconstructions in the future and, as such, *C. albarenicolus* would be an important taxon to consider in any biogeographic context (St Laurent et al. 2018, 2020b). Mimallonidae are exceedingly species-poor in North America, and any evidence of ancient colonization and persistence in unique habitats, like *C. albarenicolus* exclusively in Florida Scrub, could help to understand the evolutionary history of these enigmatic taxa in North America. Regarding the colonization of Florida, it is noteworthy that white sand scrubs, relative to other scrubs in the state, are thought to be the oldest scrubs in Florida (Myers and Ewel 1990). This suggests that *C. albarenicolus* could be a relictual taxon associated with this ancient habitat since it is apparently absent from younger yellow sand scrub. Furthermore, the inferred relative placement of *C. albarenicolus* as sister to *C. melsheimeri*, whose range includes Mexican and eastern North American, including Floridian populations, suggests that the ancestor of modern *C. albarenicolus* may have colonized Florida long before the arrival of the ancestor of *C. melsheimeri* via other routes.

We also reveal notable differences in within-group mean genetic distance when comparing *C. melsheimeri* and *C. albarenicolus*. *Cicinnus melsheimeri* has a large range, including most of the eastern United States, southern Canada, the Rocky Mountains, and central Mexico. Among our samples (which encompass much of this range), the within-group mean genetic distance is lower (1.27%) than the within-group mean genetic distance of *C. albarenicolus* from within Florida (1.87%). There appears to be some geographic structure in the barcodes of *C. albarenicolus*. The inclusion of a recent specimen from Ocala National Forest (the holotype) is recovered as sister to a 1964 specimen from geographically nearby Cassia, whereas barcodes from farther sites are more genetically distant. This result is not unexpected considering the relative isolation of individual examples of white sand scrub across Florida. Unfortunately, the potential extirpation of most populations of *C. albarenicolus* means much of this genetic diversity may be lost.

The present study’s goal was to describe and name this rare species, and for that, only mitochondrial barcodes were sequenced. Including *C. albarenicolus* in a nuclear, phylogenomic context would therefore be desirable to better understand the phylogeny and, eventually, biogeography of *Cicinnus* generally.

Now that there is formal recognition of the existence of a rare *Cicinnus* endemic to Florida, it is possible to delve further into its biology and conservation. Future work should focus on identifying extant populations of *C. albarenicolus* to better inform conservationists in the region, since clearly the species is of significant concern and may warrant protection. For context, Florida Scrub has undergone severe degradation and outright loss: 85% of the Lake Wales Ridge scrub is destroyed, and coastal scrubs (like the Port Sewall locality that formerly supported *C. albarenicolus*) are 90% destroyed (The Nature Conservancy & The University of Florida Geoplan Center 2005).

To better understand the extent to which *C. albarenicolus* still occurs, targeted collecting from sites known to have historically harbored this species, or from unsampled white sand scrub habitat, particularly in the early spring, is encouraged. Efforts should also be made to identify the food plant, larval activity period, and aspects of sexual isolation from the sympatric and much more common and widespread *C. melsheimeri*. Apart from the expectation that it is highly specialized in habitat, it is not clear why *C. albarenicolus* is so restricted since presumed oak host plant species are either more widespread than the moth, or the moth occurs in regions where different oak species occur (that is, it does not occur exclusively within a specific oak’s range). By identifying the specific factors that limit its distribution it may then be possible to better understand how to protect this species.

## Supplementary Material

XML Treatment for Cicinnus albarenicolus

XML Treatment for Cicinnus melsheimeri

## Additional information

### Use of AI

The following AI tools were used in the preparation of this manuscript: Latitude/longitude conversion, code organization.

### Author ORCIDs

Ryan A. St Laurent https://orcid.org/0000-0001-6439-5249

### Data availability

All of the data that support the findings of this study are available in the main text or Supplementary Information.

## Supplementary materials

Supplementary material 1 Decimal degrees latitude/longitude Data type xlsx Explanation note Decimal degrees latitude/longitude are used for generating the map in Fig. 24. Most records are from iNaturalist and are reported as generated natively on the website. Those from the material examined show the estimated latitude/longitude using Google Maps based on label data, when not explicitly given on the labels. https://binary.pensoft.net/file/1566911This dataset is made available under the Open Database License (http://opendatacommons.org/licenses/odbl/1.0/). The Open Database License (ODbL) is a license agreement intended to allow users to freely share, modify, and use this Dataset while maintaining this same freedom for others, provided that the original source and author(s) are credited. Ryan A. St Laurent, Scott Wehrly, Jeffrey Slotten

Supplementary material 2 Metadata Data type xlsx Explanation note Complete metadata including sample ID, process ID, GenBank accession number, collecting information and storing institutions for all mimallonids that were either newly sequenced for this study or downloaded from BOLD. https://binary.pensoft.net/file/1566912This dataset is made available under the Open Database License (http://opendatacommons.org/licenses/odbl/1.0/). The Open Database License (ODbL) is a license agreement intended to allow users to freely share, modify, and use this Dataset while maintaining this same freedom for others, provided that the original source and author(s) are credited. Ryan A. St Laurent, Scott Wehrly, Jeffrey Slotten

Supplementary material 3 FASTA alignment Data type fas Explanation note FASTA alignment used to infer the tree in Fig. 23. https://binary.pensoft.net/file/1566913This dataset is made available under the Open Database License (http://opendatacommons.org/licenses/odbl/1.0/). The Open Database License (ODbL) is a license agreement intended to allow users to freely share, modify, and use this Dataset while maintaining this same freedom for others, provided that the original source and author(s) are credited. Ryan A. St Laurent, Scott Wehrly, Jeffrey Slotten

Supplementary material 4 Newick-formatted tree file Data type tre Explanation note Newick-formatted tree file of the tree in Fig. 23. https://binary.pensoft.net/file/1566914This dataset is made available under the Open Database License (http://opendatacommons.org/licenses/odbl/1.0/). The Open Database License (ODbL) is a license agreement intended to allow users to freely share, modify, and use this Dataset while maintaining this same freedom for others, provided that the original source and author(s) are credited. Ryan A. St Laurent, Scott Wehrly, Jeffrey Slotten
